# Polarisation-sensitive optical coherence tomography measurement of retardance in fibrosis, a non-invasive biomarker in patients with systemic sclerosis

**DOI:** 10.1038/s41598-022-06783-7

**Published:** 2022-02-21

**Authors:** E. J. Marjanovic, V. Sharma, L. Smith, C. Pinder, T. L. Moore, J. B. Manning, G. Dinsdale, M. Berks, V. L. Newton, S. Wilkinson, M. R. Dickinson, A. L. Herrick, R. E. B. Watson, A. K. Murray

**Affiliations:** 1 Musculoskeletal Research Group, Salford Care Organisation, part of the Northern Care Alliance NHS Foundation Trust, Rm C215, Clinical Sciences Building, Salford, M6 8HD UK; 2 Salford Care Organisation, part of the Northern Care Alliance NHS Foundation Trust, Manchester Academic Health Science Centre, Salford, M6 8HD UK; 3 Centre for Dermatology Research, University of Manchester, Manchester, M13 9PL UK; 4 School of Physics and Astronomy, University of Manchester, Manchester, M13 9PL UK; 5 Centre for Imaging Sciences, Division of Informatics, Imaging and Data Sciences, University of Manchester, Manchester, M13 9PL UK; 6 Photon Science Institute, University of Manchester, Manchester, M13 9PL UK; 7 NIHR Manchester Biomedical Research Centre, University of Manchester, Manchester, M13 9PL UK; 8 Michigan State University College of Human Medicine, Lansing, MI USA

**Keywords:** Biotechnology, Biomarkers, Rheumatic diseases, Imaging and sensing

## Abstract

Polarisation-sensitive optical coherence tomography (PS-OCT) offers a novel, non-invasive method of assessing skin fibrosis in the multisystem disease systemic sclerosis (SSc) by measuring collagen retardance. This study aimed to assess retardance as a biomarker in SSc. Thirty-one patients with SSc and 27 healthy controls (HC) underwent PS-OCT imaging. ‘Skin score’ was assessed by clinical palpation (0–3 scale). A subset of ten patients and ten age/sex-matched HC had a biopsy and longitudinal imaging. Histological assessment included quantification of epidermal thickness, collagen content (to assess fibrosis) and matrix metalloproteinase (MMP) activity (in situ zymography). PS-OCT images were assessed for epidermal thickness (structure) and fibrosis (retardance). Positive correlation was observed between epidermal thickness as measured by histology and structural PS-OCT (r = 0.79; p < 0.001). Retardance was: HC mean 0.21 (SD 0.21) radian/pixel; SSc skin score 0, 0.30 (0.19); skin score 1, 0.11 (0.16); skin score 2, 0.06 (0.12); skin score 3, 0.36 (0.35). Longitudinal retardance decreased at one-week across groups, increasing at one-month for HC/skin score 0–1; HC biopsy site retardance suggests scarring is akin to fibrosis. Relationships identified between retardance with both biopsy and skin score data indicate that retardance warrants further investigation as a suitable biomarker for SSc-related fibrosis.

## Introduction

Systemic sclerosis (SSc) is a rare autoimmune connective tissue disease that causes changes to microvascular architecture and function, in addition to fibrosis of soft tissue. Although rare (affecting around 6000 people in the UK), SSc carries a high mortality due to the fibrosis of internal organs, and has a huge impact on those affected in terms of pain and disability^1^. There is currently no proven effective disease-modifying therapy for SSc; clinical trials have been hampered by lack of both objective clinical outcome measures and lack of biomarkers to enable the evaluation of new therapies^2,3^. Skin changes reflect disease progression and the severity of internal organ involvement, and the degree of skin thickening is a predictor of mortality^4^. In clinical practice the standard method of assessing skin involvement in patients with SSc is by modified Rodnan skin score (mRSS) determined by clinical palpation^5–7^. Although quick and simple, the mRSS is subjective and affected by oedema; studies have shown that the repeatability of the measure—even by experienced investigators—can be poor^8^. More reliable measures that are sensitive to change are therefore required. High frequency ultrasound (measuring skin thickness and oedema) and other non-invasive imaging techniques such as durometry (measuring skin hardness), plicometry (measuring thickness of skin folds) and cutometry (measuring elasticity) have been trialled but have not met all of the validity criteria that would make them usable and reliable methods of assessment. Histology remains the gold standard to assess fibrosis but has the disadvantage of being invasive^8^.

Optical coherence tomography (OCT) takes non-invasive, in vivo*,* ‘optical biopsy’ images of the skin, analogous to ultrasound, but using light to visualise skin structure^9^. This allows high resolution (< 10 µm) measurement of epidermal thickness. The reviews by Olsen et al. and Guida et al. offer excellent overviews on the wider application of OCT in dermatology and the comparison of OCT and other optical imaging techniques in skin^10,11^.

Polarisation-sensitive optical coherence tomography (PS-OCT) offers a novel non-invasive method of assessing skin fibrosis in SSc. The fibrillar collagens, a key component of the dermal extracellular matrix, cause skin to be anisotropic and thus to have birefringent properties (i.e. skin has different refractive indices depending on the polarisation state of the light, causing one light polarisation state to travel faster through the tissue than the other). Birefringence is measured as ‘phase retardation’ and can be assessed using polarised light. The retardance of a material is the change in the phase angle as the light travels through and is generally shown as cumulative colour maps of in depth images.

Polarised light transmission microscopy has been shown to be sensitive to the spatial orientation pattern of collagen in histological sections. In skin with burn damage and scarring, changes to the birefringent properties of the skin can be observed^12–15^. Collagen fibrils are denatured by burn damage. This affects the structure of the fibrils (akin to unravelling), their orientation and density. Birefringence increases once tissue repairing/scarring begins. These changes in birefringence can be used as a measure of damage and potentially fibrosis. PS-OCT allows for measuring these changes and heterogeneity within the skin in addition to skin structure. In addition, recent advancements allow further understanding of skin structure^16,17^.

This study aimed to test the hypothesis that PS-OCT could measure differences in skin thickness and fibrosis, non-invasively, between patients with a range of clinical severities of disease. The aim of this study was to gauge the potential of PS-OCT as a tool to quantify collagen retardance as a tractable biomarker of disease progression in SSc. Specific objectives were: (1) to obtain in vivo PS-OCT images of forearm skin in patients with SSc and in healthy controls, allowing comparison of structural (epidermal thickness) and retardance data (a measure of fibrosis), to the current clinically-validated method of assessment, mRSS (clinically accepted non-invasive measurement); (2) to validate in a matched subset, the PS-OCT images, comparing to biopsy tissue allowing assessment of epidermal thickness, fibrillary collagen content and potential involvement of matrix metalloproteinases (MMPs) in any observed tissue remodelling, and; (3) in a subset of those biopsied, carry out follow-up imaging to assess scarring, analogous to fibrosis.

## Patients and methods

Participants attended for a single imaging visit with the exception of a subset of ten HCs and ten patients with SSc who underwent a biopsy and were invited to attend for two follow-up visits to image the biopsy site. Participants were asked to refrain from caffeine and nicotine for four hours prior to the study and to attend without creams or make-up on the area to be imaged (the volar forearm). The study was approved by Greater Manchester West Research Ethics Committee (16/NW/0023). All research was performed in accordance with relevant guidelines and regulations. All participants gave informed, written consent for the study.

### Participants

Fifty eight participants (31 patients with SSc and 27 HC) were recruited to the study; demographics (age, gender) of all participants and the biopsy subset (ten HCs and ten patients with SSc) are shown in Table 1. In addition, for patients, duration of Raynaud’s phenomenon, duration of SSc (from onset of first non-Raynaud's clinical manifestation) and SSc subtype were recorded. Raynaud’s phenomenon (cold hands with skin colour changes) is often the first symptom of SSc. SSc has two subtypes; limited cutaneous and diffuse cutaneous, dependent upon the extent of skin thickening^18^.

**Table 1:** Demographic details of participants.

	Full cohort		Biopsy subset	
	Patients (n = 31)	HC (n = 27)	Patients (n = 10)	HC (n = 10)
**Age**				
Mean (SD) years	62 (11)	50 (9)	53 (12)	53(12)
**Gender**				
Female, n (%)	21 (68)	17 (63)	7 (70)	7 (70)
**SSc duration (years)**				
Mean (SD)	16 (10)	N/A	11 (9)	N/A
Raynaud’s phenomenon duration (years)*				
Mean (SD)	24 (16)	N/A	13 (11)	N/A
**Skin score**				
0	19	N/A	3	N/A
1	5		3	
2	4		3	
3	3		1	
**SSc subtype**				
Limited cutaneous SSc, n(%)	8 (26)	N/A	4 (40)	N/A
Diffuse cutaneous SSc, n(%) [Le Roy]	23 (74)		6 (60)	

*Raynaud’s phenomenon, cold hands with colour changes is often the first symptom of SSc.

### Skin score

All participants with SSc underwent assessment of skin thickening at the site to be imaged. Skin is scored 0–3 via clinical palpation (0 [unaffected skin], 1 [thickened; can pinch], 2 [thickened; cannot pinch but can move], 3 [thickened cannot pinch or move]^5–7^ (Table 1).

### PS-OCT image collection

**Figure 1:**
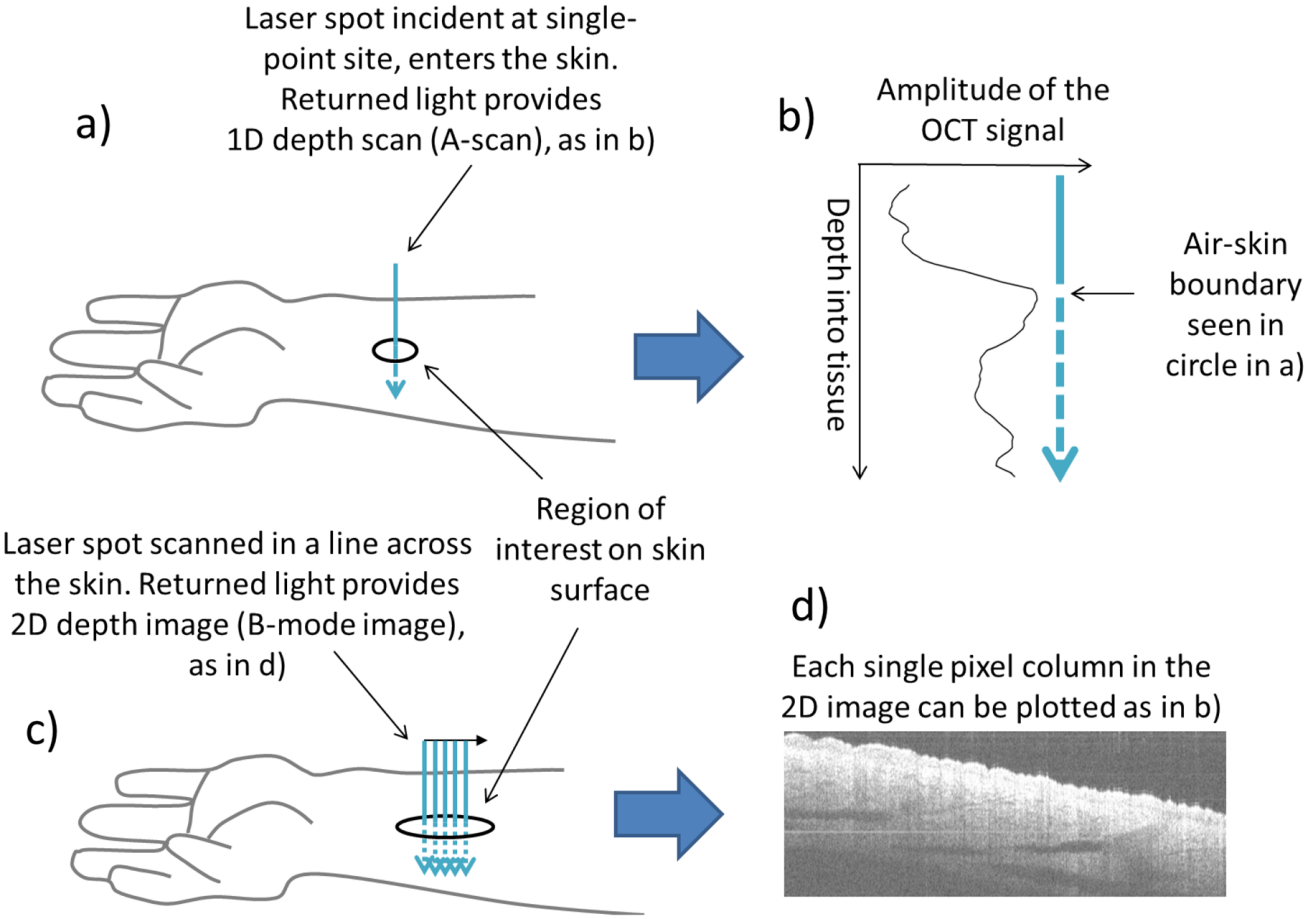
(**a**,**b**) Generation of single depth scans (A-scans). At each point an A-scan (a one-dimensional, single pixel scan into the depth of the tissue) is generated for both images (structural and retardance, here shown as the greyscale structural image). In the structural image this represents the relative amplitude of returned light (analogous to echo location in ultrasound) from each boundary encountered where refractive index changes of the tissues occur (observed as hyper- or hypo-reflective structures, i.e. peaks or troughs in the A-scan). For the retardance image the A-scan shows the relative change in birefringence with depth (birefringence induced phase-retardation [i.e. the way the light is slowed in the tissue due to fibrotic changes which alter its path]) and as the laser line scanned over the surface of the skin multiple A scans (1D images) are obtained producing a B-Mode image (x–z plane [a 2D image along a line of the skin and into the depth into the skin); (**c**,**d**) 2D depth images (B-mode scans) at the volar forearm. The black oval in (**a**) and (**c**) represents the site of imaging on the surface of the skin. The mean of the A-scans across the B-mode image was then calculated to produce a mean depth scan. The image in (**d**) (8.00 × 4.87 mm) is made up of consecutive A-scans 4.87 mm deep. Image d was generated in ThorImage 4.3.

All participants were scanned using PS-OCT (Fig. 1); a small optical scanning head (approx. 2 × 2 cm^2^) was placed on the skin of the volar forearm. This held the imaging optics at the correct distance from the skin for the surface to be in focus. There was no cover glass used, to avoid compression of the tissue within the imaging area. The system was a Thorlabs OCS1310V1/PSOCT-1310V1 (Thorlabs Inc. NJ, US; central wavelength 1310 nm, tuning bandwidth 100 nm, 16 kHz acquisition rate, linearly polarised, pixel dimension of 3.91 µm width by 9.51 µm depth). The PS-OCT captured simultaneous structural and retardance images (512 × 2048 pixels). The subset of 20 participants (Table 1) undergoing biopsy were invited to return for imaging of the site at one-week (stitch removal, visit 2) and one-month (visit 3).

### PS-OCT image analysis

**Figure 2:**
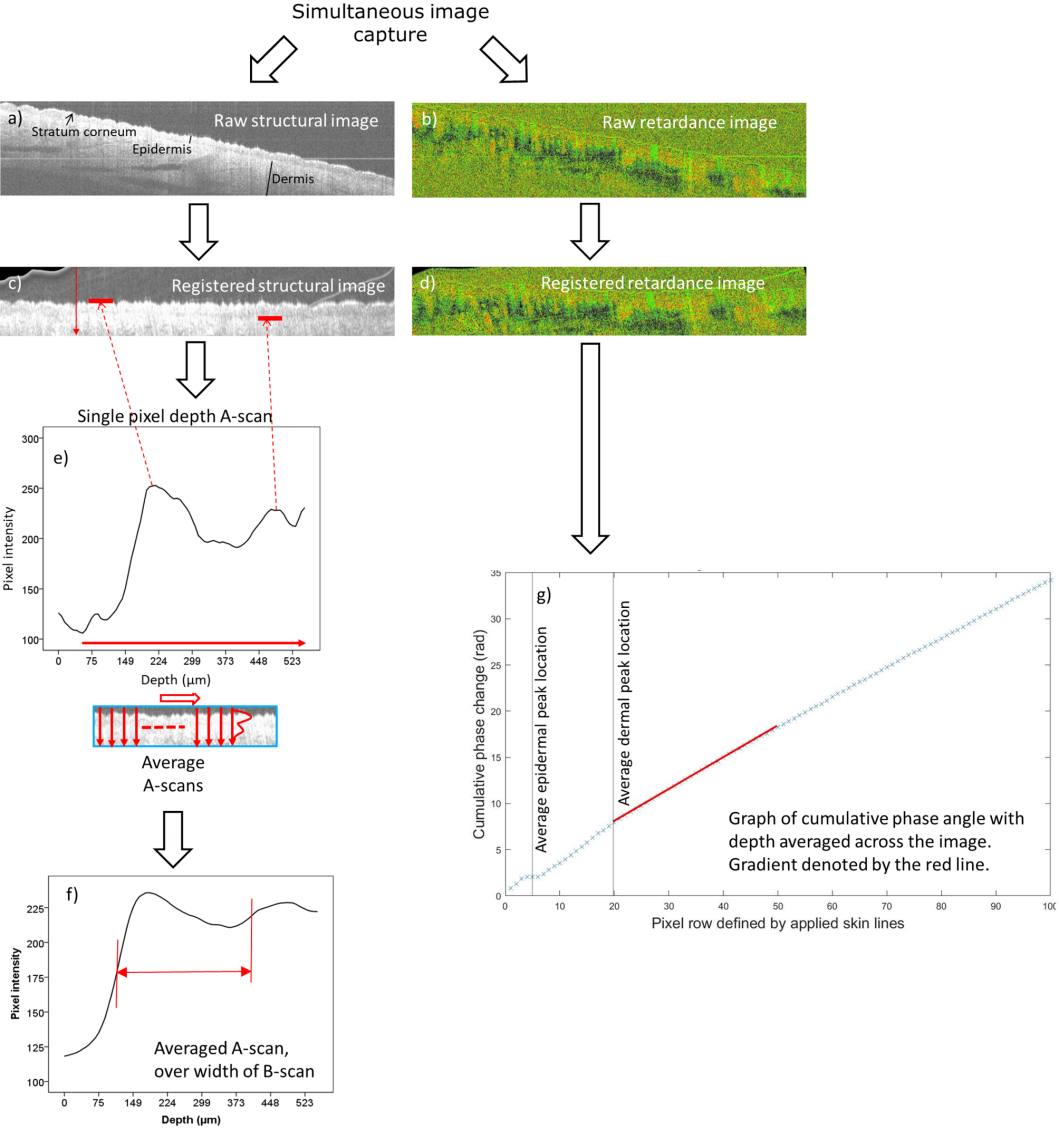
Image post-processing pathway. Examples of: raw PS-OCT B-scans for (**a**) structural (showing the layers of the skin beneath the surface) and (**b**) retardance images (birefringence manifests itself as observable skin heterogeneity and tissue organisation, heterogeneity here is due to the presence of structures such as sebaceous glands and hair follicles). Images (**a**) and (**b**) were generated in ThorImage 4.3; (**c**,**d**) Scans are registered to align slices and provide a horizontal skin surface. Registration is performed by smoothing each A-scan in the structural image and selecting the first peak as the skin-air boundary. The vertical alignment of each A-scan in the structural and birefringence images are then adjusted so the skin-air boundary lies in a horizontal line across the image (using bespoke software (written by the authors) in MATLAB (version R2020a, Mathworks Inc, Mass, USA)). This makes birefringence (retardance) analysis easier as rows of pixels in the registered images correspond to tissue of consistent depth beneath the skin. (**e**) A-scan [single pixel width depth profile] and (**f**) averaged A-scan taken across the B-mode image [i.e. averaging all the A-scans over the width of the image to give an averaged measure of where peaks reflected from layers with in the skin occur]. Epidermal thickness was defined as the distance between the depth at which the intensity of the first peak had reached half its maximum value (skin surface) and the depth at which the intensity of the second peak had reached half its maximum value (dermal–epidermal junction); marked as a red horizontal arrow. (**g**) Shows the cumulative retardance (phase change [radians]) plotted for skin depth (pixels) from skin surface to 100 pixels (shown without errors). The epidermal and dermal peak locations are shown as grey vertical lines. The gradient at the first 30 pixels from the dermal line (used to measure the differences between the groups) is indicated by the red line.

Images were extracted using ThorImage 4.3 acquisition software (part of the Thorlabs system). Structural (intensity) images were analysed using ImageJ (National Institutes of Health, MA, USA version 1.48). Retardance images were analysed using bespoke software (written by the authors) in MATLAB (version R2020a, Mathworks Inc, Mass, USA). The capture and processing pathway is shown in Fig. 2.

In ImageJ the structural images underwent post-capture processing, including ‘flattening’ the images by registering (aligning) the pixels of the surface of the skin horizontally. The greyscale values in the structural images represent the reflected amplitude of light from each boundary within the skin. As shown in Fig. 2, measurements of epidermal thickness were performed on averaged ‘A-scans’ (amplitude of a single line of pixels into the skin) taken across the greyscale structural images mean structural depth scan. Epidermal thickness was defined as the distance between the depth at which the intensity of the first peak from the A-scan had reached half its maximum value (maximum signal occurs at the skin surface) and the depth at which the intensity of the second peak had reached half its maximum value (dermal–epidermal junction), as per previous studies^19,20^. It should be noted that the exact location of the dermal–epidermal junction in PS-OCT structural images was slightly ambiguous due to the boundary being neither thin, nor flat, but composed of rete ridges which could not be individually resolved by this PS-OCT system^21^. It has been shown previously that in patients with SSc the boundary becomes less identifiable with increasing skin score^22^.

Retardance images (each pixel representing phase retardation) were further processed and analysed in MATLAB. Retardance images were registered (using the same process as for structural images, since the PS-OCT and structural images are intrinsically co-registered) to account for surface roughness. Phase retardation measurements above the assigned skin line were disregarded and the remaining data set was unwrapped. This produced an array of cumulative phase change. Images were then averaged across the width of the area of interest. It is commonly accepted that birefringence in the region between the epidermis and dermis layers is weak, not inducing local retardance^17^. Thus retardance was assessed from the epidermal-dermal junction to 100 pixels below (where the signal from the skin is lost). In order to ease comparison between images the minimum rate of change recorded for each image was used as a reference value to zero the minimum rate of cumulative phase change in this region of each image. Images were then averaged together within assigned groupings and plotted. The gradient of these curves was then calculated by least squares fit from the dermal peak to 30 lines below (approximately 285 µm; based upon observation of when the gradient started to become non-linear).

### Biopsy collection

A 4 mm punch biopsy was obtained from a subset of the participants; ten patients with SSc (including different grades of skin score) and ten age- and sex-matched HCs. The biopsy was taken at the same location as the PS-OCT scan, the skin of the volar forearm. Biopsies were bisected. One half was embedded in optimal cutting temperature compound (Miles; IN, USA) and flash-frozen in liquid nitrogen; the remaining half was fixed in buffered formalin and processed to wax (formalin-fixed, paraffin-embedded, [FFPE]).

### Biopsy histology analysis

FFPE samples were cut to sections 5 µm thick. Standard histology samples were stained with haematoxylin and eosin (H&E) to identify epidermal thickness (under standard light microscopy). Picrosirius red (PSR) staining was performed to identify fibrillar collagen content (brightfield) and organisation (under polarised light). Percentage collagen was calculated in brightfield; the dermal tissue area was measured by image analysis (a free-drawing tool) prior to measuring the area positive for collagen; these data were used to calculate the percentage of dermal matrix occupied by fibrillar collagens.

To assess any potential collagen remodelling in the tissue, frozen sections were interrogated using in situ zymography to assess the activity of MMP-1 (collagenase) and MMPs-2 & -9 (72 kDa and 92 kDa gelatinases), essential enzymes for the remodelling of collagenous extracellular matrices (markers measuring synthesis/deposition and remodelling).

Relationships between epidermal thickness (from PS-OCT and histology), retardance gradient and percentage collagen and collagenase and gelatinase activity were assessed by Spearman’s correlations (Stata v14.0, StataCorp, Tx, US). Despite sample size, formal statistics comparing groups were carried out (Kruskal Wallis test); however, it is important to note that given the small sample size (when patients are in subgroups), absence of statistical significance cannot be interpreted to be due to the lack of relationships. Due to the small numbers in the SSc subsets, for plots comparing cumulative retardance with skin depth, patients with SSc were grouped in to skin score 0–1 and skin score 2–3 for comparison to the HC group.

## Results

Comparison of epidermal thickness to the current clinical method of assessment, skin score, shows increased epidermal thickness with increasing skin score (Table 2). Phase retardation in patients with SSc is more variable and decreases in skin scores 1 and 2 before increasing in skin score 3. However, when grouped into HC, skin scores 0–1 and 2–3 there is a trend for increased retardance with increasing fibrosis (Fig. 4a).

**Table 2:** Mean (SD) values of PS-OCT and histology.

Skin score for SSc group for whole cohort PS-OCT imaging [SSc n = 31; HC n = 27]		HC	SSc			
		NA	Skin score 0 [n = 19]	Skin score 1 [n = 5]	Skin score 2 [n = 4]	Skin score 3 [n = 3]
PS-OCT Structural image	Epidermal thickness (µm)	251.02 (25.08)	259.92 (28.31)	271.71 (34.64)	290.39 (60.59)	355.49 (65.98)
PS-OCT Retardance [SSc n = 31; HC n = 26 ]	Gradient of phase angle change (radian/pixel)	0.21 (0.21)	0.30 (0.19)	0.11 (0.16)	0.06 (0.12)	0.36 (0.35)

Skin score for SSc group for histology/zymography subset [SSc n = 10; HC n = 10]		NA	Skin score 0 [n = 3]	Skin score 1 [n = 3]	Skin score 2 [n = 3]	Skin score 3 [n = 1]
Histology	Epidermal thickness (µm)	33.87 (6.18)	30.98 (9.40)	45.29 (17.16)	53.14 (31.96)	67.18 No SD (n = 1)
Histology	Percent collagen in dermis (%)	26.46 (6.47)	23.39 (4.82)	16.48 (8.38)	19.42 (9.97)	37.28 No SD (n = 1)
Zymography	MMP-1 (collagenase, arb units)	467.25 (66.43)	506.93 (42.76)	424.22 (51.92)	391.25 (80.35)	458.91 No SD (n = 1)
Zymography	MMPs-2, -9 (gelatinase, arb units)	799.46 (195.87)	1024.96 (114.71)	877.23 (167.34)	782.10 (393.41)	698.67 No SD (n = 1)

### Imaging epidermal thickness

**Figure 3:**
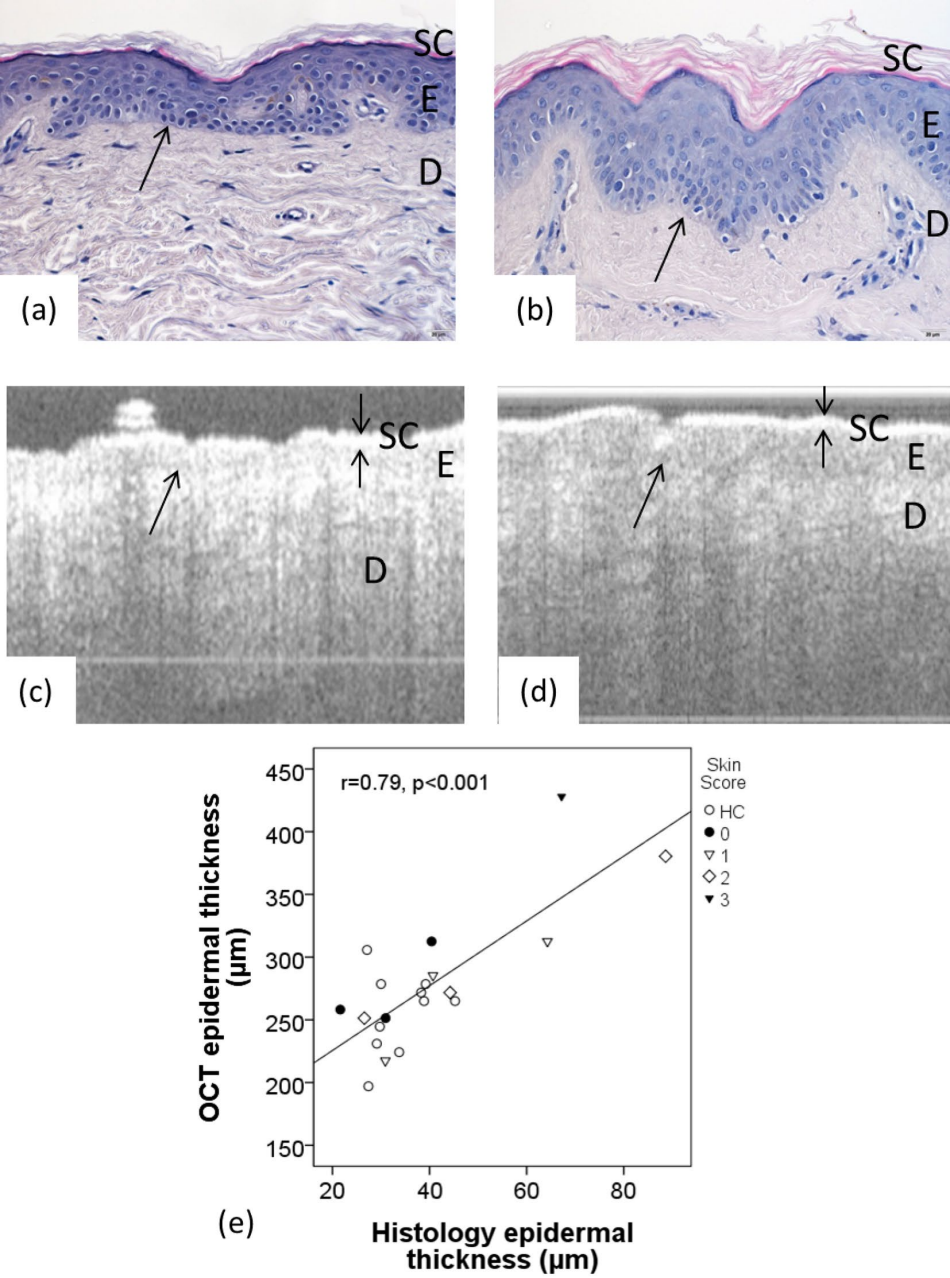
H&E stained histology showing skin thickness in: (**a**) a HC and; (**b**) a patient with SSc (skin score = 3). Structural PS-OCT in: (**c**) the same HC and; (**d**) same patient with SSc. Skin structure is labelled as *stratum corneum* (SC, indicated by double arrow in (**c**) and (**d**), epidermis (E), dermis (D) and the single arrow shows the dermal–epidermal junction; scale bar 20 µm. (**e**) displays the positive correlation between histology and PS-OCT (r = 0.79, p < 0.001; n = 20), for HC and patients, patient skin score shown on the legend as 0–3.

Epidermal thickness as measured by both classical histology and by structural PS-OCT and was found to be greater in the SSc cohort with skin scores 1–3 than the control group, regardless of the method of assessment (Table 2, examples shown in Fig. 3a–d). There was a trend for increased epidermal thickness with increasing skin score in the patient group.

### Retardance data

**Figure 4:**
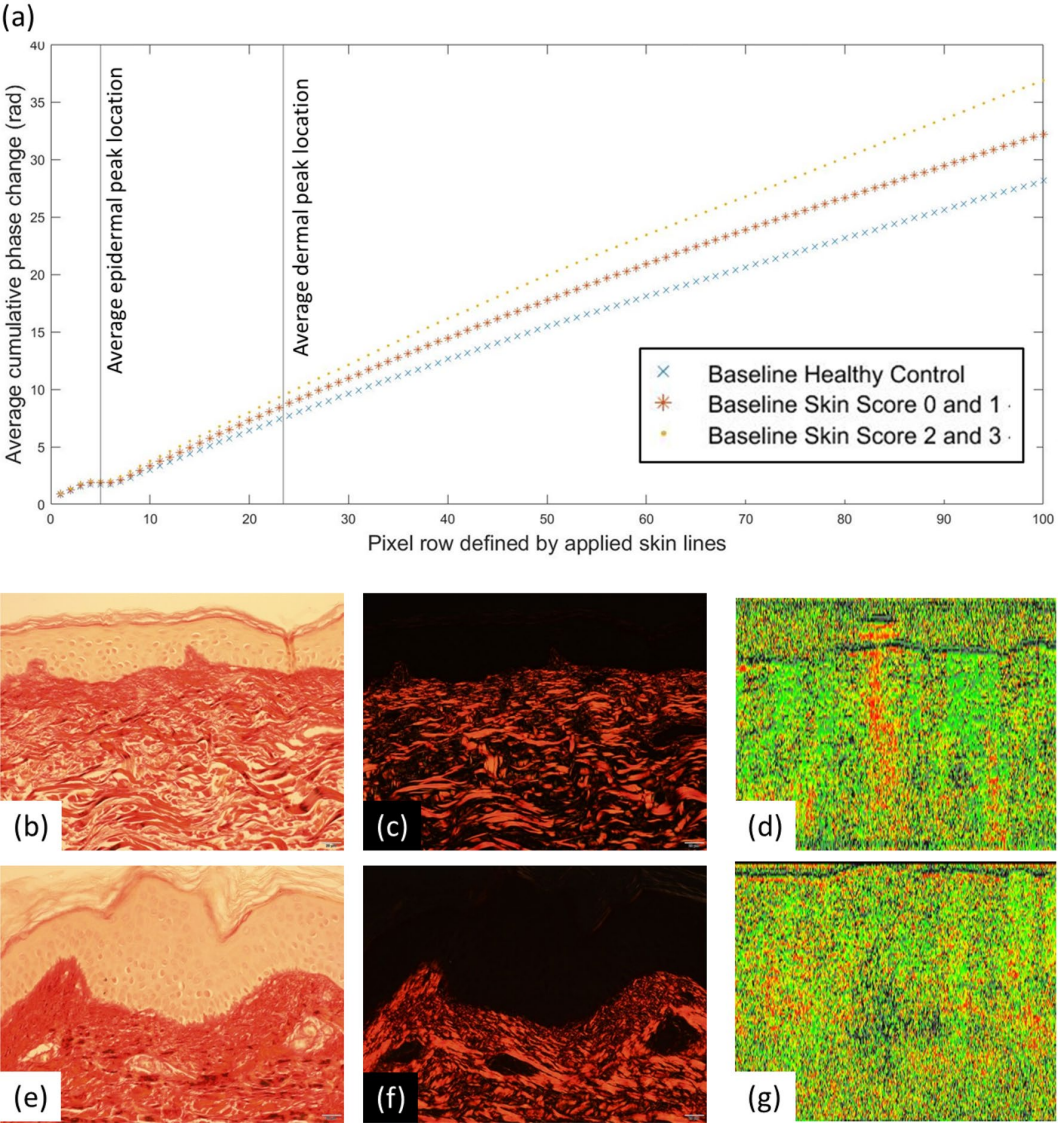
(**a**) Plot of baseline cumulative retardance with depth for HCs (N = 26) and patients with SSc grouped according to 0–1 (N = 24) and 2–3 (N = 7) skin score (shown without error bars, graphs with error bars provided in Supplemental data). Averaged locations for the epidermal and dermal peaks are denoted by the vertical grey lines. The graph indicates a higher gradient for patients with skin score 2–3 as compared to those with 0–1 and HCs. (**b**) PSR brightfield histology, skin score = 0–1; (**c**) PSR polarised histology, patient skin score = 0–1; (**d**) PS-OCT matching retardance images (arbitrary false colour); (**e**–**g**) corresponding imaging techniques in a patient with SSc (skin score = 2–3). Images (**d**) and (**g**) were generated in ThorImage 4.3.

The gradient of the phase angle change with skin depth is shown in Table 2 and, for grouped data, in Fig. 4a. Retardance images from one of the 27 controls could not be opened in the bespoke software. Gradient phase change did not differ significantly between HCs and patients (Kruskal Wallis; p = 0.54), nor between groups of skin scores (Kruskal Wallis; p = 0.10); although the small samples size must be taken into account when interpreting these data. When grouped (Fig. 4a), the gradient was lower in HCs than those with skin score 0–1 (mild fibrosis) who had in turn a lower gradient than those with skin score 2–3 (moderate/severe fibrosis); i.e. retardance was higher in those with fibrosis.

### Comparison, in a matched subset, of the PS-OCT to biopsy tissue identified relationships between epidermal thickness and matrix metalloproteinases (MMPs)

#### Imaging epidermal thickness

Statistically significant correlation was observed between epidermal thickness as measured by histology and structural PS-OCT (r = 0.79, p < 0.001; Fig. 3e).

#### Biopsy data

In situ zymography showed a trend for MMP activity to be reduced in SSc biopsy tissue with increasing skin score, suggesting that skin-resident enzymes may have been utilised to facilitate remodelling in line with increasing skin score (Table 2). Percentage collagen, identified by PSR staining, decreased with increasing skin score = 0–2 but was increased in skin score = 3 (Table 2).

#### Retardance (phase retardation) data

No relationship was found between optical retardance gradients and percentage collagen identified by PSR (0.23, p = 0.340), or MMP activity in the participants that underwent biopsy (gelatinase − 0.14, p = 0.548; collagenase − 0.27, p = 0.257). Images from biopsy and retardance PS-OCT are shown in Fig. 4b–g.

### The impact of the biopsy on scarring is akin to fibrosis and relates to changes in retardance

**Figure 5:**
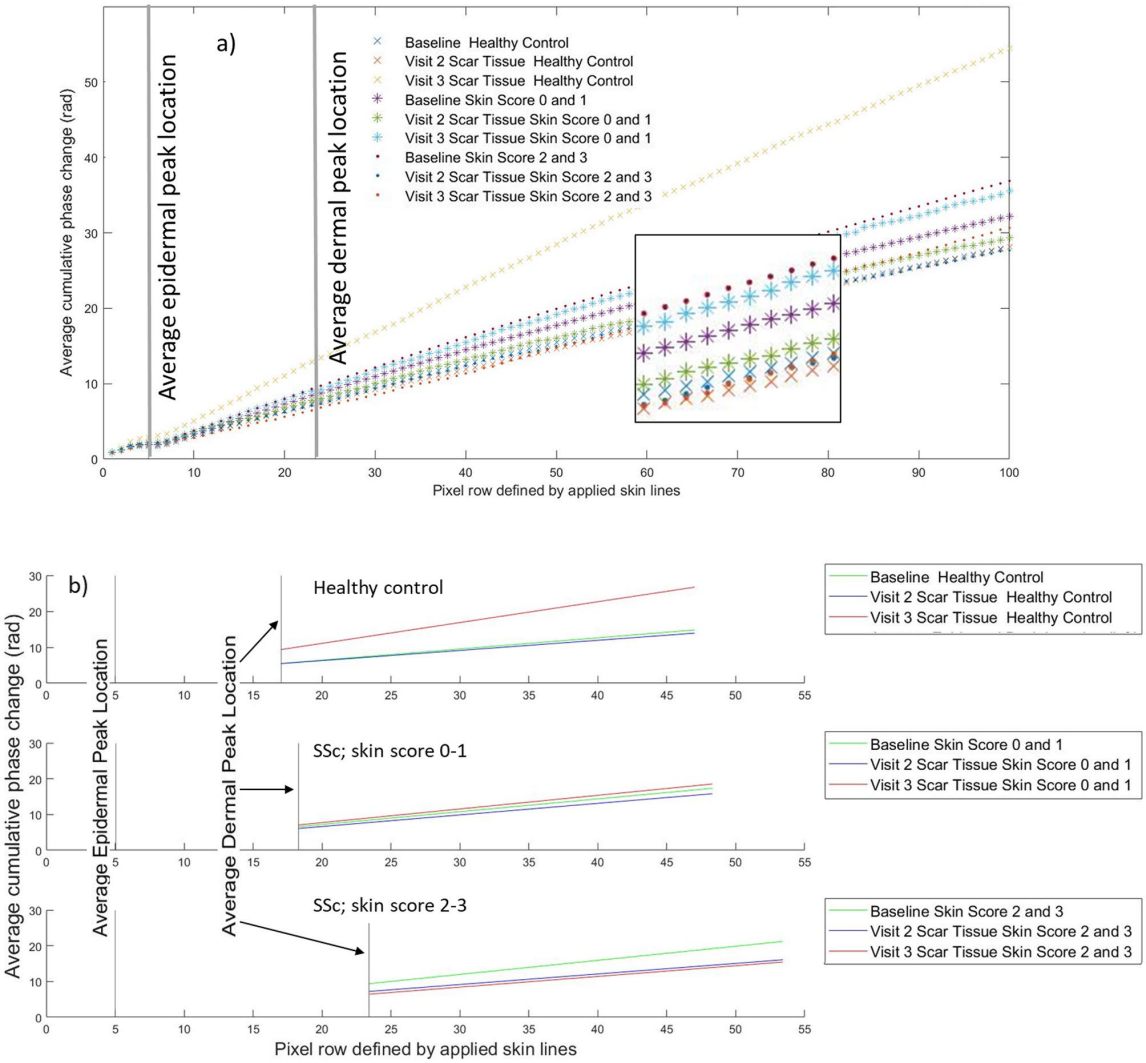
(**a**) Cumulative retardance plotted with depth for the three visits grouped for HCs and patients with skin scores 0–1 and 2–3 (without error bars for clarity, a version with error bars is shown in the Supplementary data). Averaged locations for the epidermal and dermal peaks over all groups are denoted by the vertical grey lines. Inset box is a higher magnification of the lines to enable clearer view of the trends. Baseline data shows the same trend as for Fig. 4. The graph indicates that for both HCs and patients with skin scores of 0–1 (mild fibrosis) retardance decreases from baseline to one-week post biopsy visits but then increases by the one-month visit to above the baseline retardance value, with the HC one-month retardance being higher than SSc values. In contrast the patients with skin score 2–3 (moderate to severe) whilst also showing a show a decrease at the one-week visit show an increase at one-month that is still below the baseline retardance value. Baseline N = 10 for both HC and SSc, one-week visit, N = 8 for HCs and N = 7 patients, one-month N = 5 for HCs and N = 4 for SSc; (**b**) gradients of the data in (**a**) displayed grouped across the baseline, 1-week and 1-month visits for each group separately; average dermal peak is shown per group. Each gradient beginning at the individual group average for dermal peak location.

The data for longitudinal follow-up visits (biopsy-scar imaging) as compared to baseline data (non-scar tissue imaging) is shown in Fig. 5 (eight HCs and seven patients attended for the one-week visit; five HCs and four patients attended for the one-month visit). Figure 5a shows all data on a single plot and 5b separates the date according to HC or SSc skin score group to allow the trends to be observed more clearly. The baseline data in the biopsy subset graph shows the same trend at baseline as in the wider group; retardance is higher with increasing skin score (comparing HC, 0–1, 2–3). For all groups retardance decreased at the one-week visit and for all but skin score 2–3 then increased at the one-month visit. For HC and groups 0–1 the increase in retardance at the one-month visit is higher than baseline. For patients with skin score 2–3 the one-month retardance did not to return to baseline values. The HC one-month retardance levels were the highest of all the groups.

## Discussion

In this study we have, for the first time in SSc, utilised both structural and retardance images from PS-OCT in vivo imaging to assess the skin of patients with SSc. When grouped, the retardance gradient (radian/pixel) was higher for patients with skin score 2–3 than for those with skin score 0–1 and these were higher than in the HC group, indicating that despite our small sample size we are able, by grouping data, to detect a relationship between retardance and fibrosis.

The structural PS-OCT image data correlated with epidermal thickness identified from biopsy tissue, confirming data shown by previous studies^22,23^. Taken together these studies, including this one, indicate that OCT can be used as a technique for measuring epidermal thickness reflective of histological samples. We did not identify a relationship between retardance and biopsy histology data. The activity of MMPs-1 (collagenase), -2 and -9 (gelatinase) in biopsies, relates to collagen remodelling and/or turnover. Collagenase was highest in HC and lower skin scores. Gelatinase was highest in skin scores 0 and 1. The full relationship between MMPs, fibrosis and retardance are yet to be fully understood.

The longitudinal retardance data decreased in all groups (HC and SSc groups 0–1 skin score and 2–3 skin score) from baseline to one-week. For HC and SSc groups 0–1 skin score retardance then increased between one-week to one-month. For HC and skin score 0–1 the one-month retardance values were higher than baseline; indicative that retardance increases with long term scarring. For 2–3 skin score (who have more severe fibrosis prior to biopsy), follow-up visits were close in value and did not return to baseline values. HC one-month retardance was higher than that of the 0–1 and 2–3 SSc skin score groups; indicating that scarring in the HC groups is similar to fibrosis observed in patients (the next highest value was the 2–3 skin score baseline value).

How these differences relate to changes in the skin needs to be further investigated. It is likely that in fibrotic tissue, the usual open basket weave arrangement of collagen fibres has been replaced with a more uniform alignment of the collagen, making the tissue less flexible; our data suggests that these changes can be directly related to the different birefringent properties of aligned bundles of collagen fibrils. Thus PS-OCT potentially provides a new non-invasive method for assessing fibrosis. These data indicate that there is no significant decrease in remodelling with SSc; but rather the balance is shifted towards greater accumulation of collagen that favours/equates to fibrosis.

The skin changes in patients with SSc are due to a combination of epidermal thickening, fibrosis and oedema, thus making it difficult to assess any one independently; PS-OCT offers the opportunity to assess all three skin properties. Whilst cutaneous fibrosis is well-described, epidermal thickening^24^ and oedema^25^ have also been described in systemic sclerosis; hence, the data presented here supports the concept that such imaging measures report these aspects of pathology in the skin of SSc patients. Here we have assessed epidermal thickness (in structural PS-OCT images) and fibrosis (in the retardance images). Oedema causes a reduction in the visibility of the boundaries by altering the refractive index of the tissue, leading to less reflection at boundaries. Previous studies have measured this as a change in optical density^22,26^). We have not assessed optical density as part of this study as oedema may be transient and not be a maintained feature of the skin of SSc patients; instead, we have focussed on novel retardance measurements to assess fibrosis. The current standard clinical method for assessing changes in skin fibrosis is the mRSS, which is subjective. Gold standard histology is invasive and as such cannot be used longitudinally. Objective, non-invasive measures are therefore highly desirable for early phase clinical studies to assess efficacy of emerging therapies for SSc.

High frequency ultrasound has been used to measure skin thickness, including in the hands and the forearm^27,28^. Forearm skin thickness has been found to be inversely correlated to disease duration in patients with SSc and increased as compared to controls^29^. Assessments have showed good inter- and intra-observer reliability and correlation to mRSS^28–30^. It has further been used to identify increased skin thickness and oedema in early disease (less than two years)^31^, including longitudinally^32^.

Other techniques for measurement of skin fibrosis in SSc have included durometry, plicometry and cutometry. Durometry has shown good inter- and intra-observer reproducibility, and is more sensitive than mRSS^33^ but, despite the change in durometry scores correlating well with the change in mRSS, there can be a wide range of durometry scores within each skin score^34^. Plicometry and cutometry have also been assessed at different anatomical sites but are not fully validated and the review by Merkel et al. notes that these techniques require further validation^33^.

Structural OCT has been used in several studies of skin outside of SSc^12–14,35–37^. PS-OCT has been used ex vivo and in vivo to compare normal skin with that of damage due to burns, hypertrophic and fibrotic scars^12,14^. It has been noted that in thermally-damaged skin collagen retardance decreased because of protein denaturation; conversely as scars are formed the re-organized collagen exhibits increased retardance relative to normal skin^37^. There are a small number of structural OCT studies specific to SSc. Abignano et al.^22^ found significant differences in the ‘optical density’ of tissue with depth in structural OCT images of 21 patients with SSc and 22 healthy controls and were able to discriminate between patients with different mRSS scores. However, OCT epidermal thickness was compared against only three biopsy samples (two patients and one healthy control). The dermal–epidermal junction could not easily be observed in the patient OCT images, indicative of optical density changes with increasing fibrosis and likely partly due to the index-matching effect of oedema in the tissue ‘washing out’ boundaries. Pires et al.^26^ have confirmed a similar finding of a relationship between optical density and mRSS as measured by OCT in a cohort of 33 Brazilian patients and 35 controls where images were taken at the finger and forearm.

We acknowledge that this study was relatively small. It was designed to collect, analyse and compare PS-OCT to skin score and histology. Our structural PS-OCT epidermal thickness measurements obtained in the control cohort were larger in magnitude than measurements of epidermis made by other studies using confocal microscopy, histology and OCT^19,35,36^. This is likely due to, in part, our choice of anatomical location (volar forearm), the choice of peaks in the A-scan used to calculate epidermal thickness and potentially the lack of visualisation of the rete ridges, due to the resolution of the system. Skin biopsies taken for histological analysis are further subject to shrinkage due to tissue contractility, fixation and processing^38^.

The PS-OCT system had a single linearly polarised input. Thus images appearance and data extracted are more reliant on sample axis orientation than for circularly polarised light. Since the scanning head allowed perpendicular positioning of the probe on the surface of the skin, the effect should have been minimal. In addition, since skin does not have a well-defined optic axis (as opposed to striated tissue such as muscle), the sensitivity of data to sample alignment should also be minimal.

Epidermal thickness, as measured by structural PS-OCT and histology, showed strong correlation (r = 0.79, p < 0.001) as per previous studies in control groups, providing robust evidence that PS-OCT can be used to quantify this anatomical feature^23,39^. The ungrouped (HC and individual skin scores 0–3) retardance gradient-skin score relationships were not statistically significant (possibly due to small sample size); however, the retardance data, when grouped, is indicative of increasing retardance with increasing skin score (fibrosis). Biopsy longitudinal data suggests that in HCs as scarring occurs retardance increases to similar levels observed in those with more severe fibrosis.

This study therefore suggests that PS-OCT may be a useful non-invasive technique for simultaneously assessing both epidermal thickness and retardance of collagen in tissue associated with fibrosis, and so may be a reliable clinical measure in longitudinal studies of patients and as an outcome measure in clinical trials. Larger studies now need to be carried out for robust validation.

## Supplementary Information

Supplementary Figures.
