# Supplementary material for: Polarisation-sensitive optical coherence tomography measurement of retardance in fibrosis, a non-invasive biomarker in patients with systemic sclerosis
Source: Sci Rep. 2022 Feb 21;12:2893. doi: 10.1038/s41598-022-06783-7 (PMC8861061; doi:10.1038/s41598-022-06783-7)
Supplement: Supplementary file 1 — Supplementary Figures. [file 41598_2022_6783_MOESM1_ESM.docx]

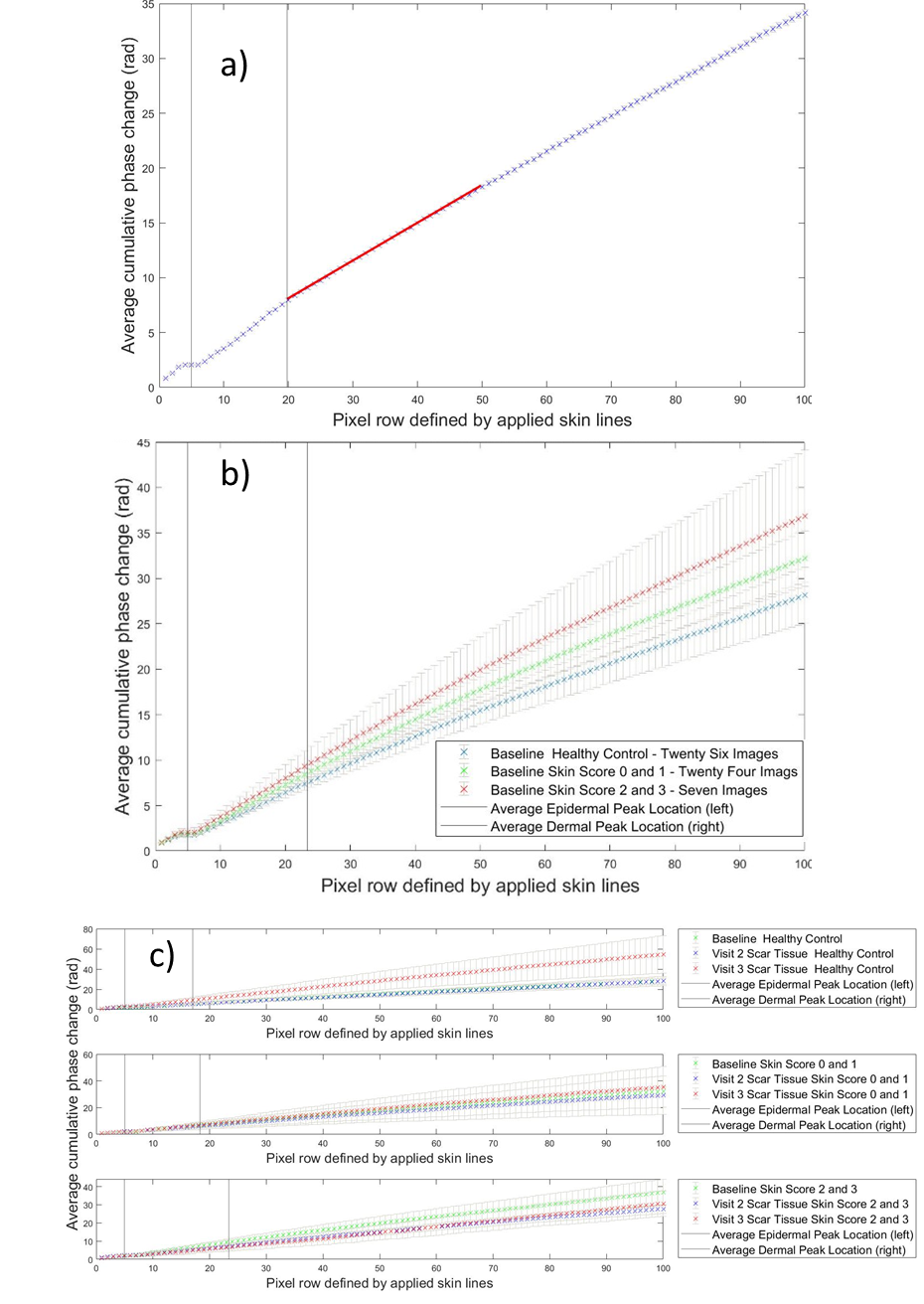


Supplemental figure: Plots from a) Figure 2g, b) Figure 4a and c) Figure 5 with standard errors (in grey) for cumulative measures in depth. In the main figures graphs are shown without errors for clarity of data observation.
